# Stressors in nursing students during the COVID-19 pandemic

**DOI:** 10.1192/j.eurpsy.2022.1287

**Published:** 2022-09-01

**Authors:** H. El Kefi, W. Kabtni, I. Bouzouita, C. Bencheikh, O. Torkhani, I. Gafsi, A. Baatout, W. Krir, A. Oumaya

**Affiliations:** Hmpit, Psychiatry, Tunis, Tunisia

**Keywords:** Stress, nurse student, stressors, Coronavirus

## Abstract

**Introduction:**

The coronavirus epidemic started in Tunisia on March 12, 2020. Nursing students in hospital internship are among the professional categories most exposed to the virus.

**Objectives:**

To identify stressors during a COVID-19 pandemic among senior nursing students at the military health school.

**Methods:**

Descriptive, retrospective study conducted in March 2021 of the 60 senior nursing students enrolled in the military health school. We developed a self-questionnaire with questions about potential stressors during a COVID-19 pandemic.

**Results:**

Our population was 54.3% male and 45.7% female. The mean age was 22.6 years. Most of the senior students (54.3%) worked in units dedicated to the care of patients with COVID-19. The main stressors reported by the students were fear of seeing patients die (84%), contaminating family (81.4%), being assigned to a COVID unit (78%), lack of protective equipment (75%), catching COVID-19 (67%), contaminating colleagues (64%), delay in teaching (61%), lack of competence and making mistakes (53%).

**Conclusions:**

The COVID-19 pandemic is a time of major stress for nursing students. Psychological support should be provided.

**Disclosure:**

No significant relationships.

